# Altered Spontaneous Brain Activity in Subjects With Different Cognitive States of Biologically Defined Alzheimer's Disease: A Surface-Based Functional Brain Imaging Study

**DOI:** 10.3389/fnagi.2021.683783

**Published:** 2021-08-30

**Authors:** Zili Zhu, Qingze Zeng, Linghan Kong, Xiao Luo, Kaicheng Li, Xiaopei Xu, Minming Zhang, Peiyu Huang, Yunjun Yang

**Affiliations:** ^1^Department of Radiology, The First Affiliated Hospital of Wenzhou Medical University, Wenzhou, China; ^2^Department of Radiology, The Second Affiliated Hospital, Zhejiang University School of Medicine, Hangzhou, China; ^3^Institute for Medical Imaging Technology, School of Biomedical Engineering, Shanghai Jiao Tong University, Shanghai, China

**Keywords:** Alzheimer's disease, A/T/N system, resting-state functional magnetic resonance imaging, amplitude of low frequency fluctuation, spontaneous brain activity, surface-based analysis

## Abstract

**Background:** Before the apparent cognitive decline, subjects on the course of Alzheimer's disease (AD) can have significantly altered spontaneous brain activity, which could be potentially used for early diagnosis. As previous studies investigating local brain activity may suffer from the problem of cortical signal aliasing during volume-based analysis, we aimed to investigate the cortical functional alterations in the AD continuum using a surface-based approach.

**Methods:** Based on biomarker profile “A/T,” we included 11 healthy controls (HC, A–T–), 22 preclinical AD (CU, A+T+), 33 prodromal AD (MCI, A+T+), and 20 AD with dementia (d-AD, A+T+) from the Alzheimer's Disease Neuroimaging Initiative (ADNI) database. The amplitude of low-frequency fluctuation (ALFF) method was used to evaluate the changes of spontaneous brain activity, which was performed in the classic frequency band (0.01–0.08 Hz), slow-4 (0.027–0.073 Hz) band, and slow-5 (0.01–0.027 Hz) band.

**Results:** Under classic frequency band and slow-4 band, analysis of covariance (ANCOVA) showed that there were significant differences of standardized ALFF (zALFF) in the left posterior cingulate cortex (PCC) among the four groups. The *post-hoc* analyses showed that under the classic frequency band, the AD group had significantly decreased zALFF compared with the other three groups, and the cognitively unimpaired (CU) group had decreased zALFF compared with the healthy control (HC) group. Under the slow-4 band, more group differences were detected (HC > CU/MCI > d-AD). The accuracy of classifying CU, mild cognitive impairment (MCI), and AD from HC by left PCC activity under the slow-4 band were 0.774, 0.744, and 0.920, respectively. Moreover, the zALFF values of the left PCC had significant correlations with cerebrospinal fluid (CSF) biomarkers and neuropsychological tests.

**Conclusions:** Spontaneous brain activity in the left PCC may decrease in preclinical AD when cognitive functions were relatively normal. The combination of a surfaced-based approach and specific frequency band analysis may increase sensitivity for the identification of preclinical AD subjects.

## Introduction

Alzheimer's disease (AD) is a major neurodegenerative disease in elderly adults that causes memory decline, executive function impairment, and dementia (Masters et al., 2015). Due to the irreversible damage of neurons, early detection and intervention may be particularly significant to reduce the damage of AD. According to the biological definition in 2018 by National Institute on Aging and Alzheimer's Association (NIA-AA), individuals with both positive biomarker profiles “A/T” (A+T+) can be diagnosed as biological AD (Jack et al., 2018). Meanwhile, according to cognitive status, they could be further classified into three groups: preclinical AD (cognitively unimpaired, CU), prodromal AD (mild cognitive impairment, MCI), and AD with dementia (dementia, d-AD) (Jack et al., 2018), representing different stages of the disease. These criteria provide an important basis for exploring early brain manifestations of the disease and developing early imaging markers.

Based on resting-state functional magnetic resonance imaging (rsfMRI), the amplitude of low-frequency fluctuations (ALFF) is a relatively reliable and reproducible method to measure local spontaneous brain activities (Zang et al., 2007; Margulies et al., 2010; Zuo et al., 2010). It has been widely used to analyze the functional differences among patients with AD, MCI, and healthy subjects. Several studies have found gradually disordered inherent activity in the brain and abnormalities of low-frequency oscillations in many brain regions mainly located in the posterior cingulate cortex (PCC), medial prefrontal cortex (MPFC), temporal regions, and superior frontal regions, suggesting that ALFF might be a potentially useful tool for detecting AD-related brain alterations (Han et al., 2011; Wang et al., 2011; Xi et al., 2012; Liang et al., 2014). Additionally, while brain activities under classic frequency bands (0.01–0.08 Hz or 0.01–0.1 Hz) were well-investigated, the inherent patterns of brain activity are sensitive to specific frequency bands (Buzsaki and Draguhn, 2004; Yang et al., 2018). It has been demonstrated that rs-fMRI signals of cortex and cistern may have different power distribution characteristics in different frequency ranges (Zou et al., 2008). Therefore, few studies divided the classic frequency band into several sub-bands and found that subjects within the AD continuum had frequency-dependent brain alterations, suggesting the unique contribution of AD pathologies (Liu et al., 2020; Yang et al., 2020).

**Figure 1:**
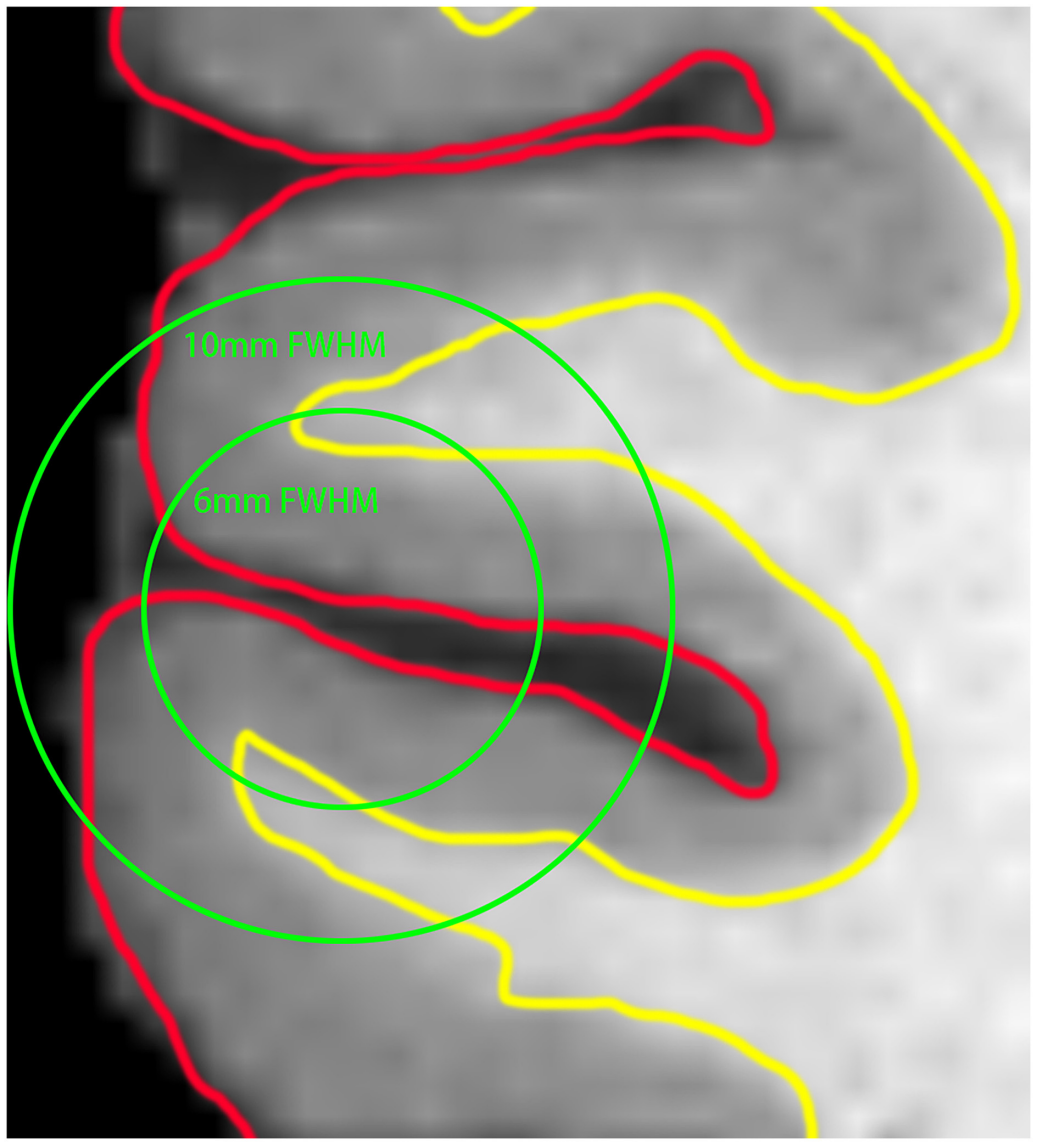
The red line represents the outermost gray matter (GM) of the brain, and the yellow line indicates the boundary between the GM and white matter (WM) of the brain. Using the full width at half maximum (FWHM) of 6 and 10 mm for voxel-based smoothing, it can be seen that the signals from GM located in different brain regions are more likely to be mixed with the increase of the smoothing kernel.

Currently, most neuroimaging studies rely on volume-based analysis to reveal the effect of disease on regional brain activities, and spatial smoothing is a necessary step to reduce the influence of noise and individual differences in brain morphology. Nevertheless, due to large smoothing kernels (6–10 mm) are usually needed, this step can lead to the aliasing of signals from the adjacent cortices with distinct functions, resulting in decreased spatial accuracy and statistical power (Figure 1). As the cortex is a large surface, it can be unfolded. By expanding the brain to the surface space, mutual contamination between neighboring but functionally distinct cortices can be avoided (Brodoehl et al., 2020). Therefore, this method has better sensitivity and more accurate spatial positioning than the traditional volume-based method in locating blood oxygenation level-dependent (BOLD) signal sources within the cortex (Andrade et al., 2001; Jo et al., 2007). Previously, there were few studies based on surface analysis because of computational difficulties (Oosterhof et al., 2011; Tucholka et al., 2012). With the enhancement of computer performance and improvement of the method, surface-based functional analysis becomes relatively practical.

The study aimed to explore the differences of surface-based spontaneous brain activity patterns in different stages of patients with AD with common biomarkers. Based on the previous studies (Liang et al., 2014; Yang et al., 2018; Zeng et al., 2019), we hypothesized that: (1) patients with biologically defined AD might have abnormal brain activities within regions related to AD pathologies, such as MPFC and PCC. (2) These changes could represent different cognitive stages and are related to cognitive functions. (3) Compared to volume-based analysis, the surface-based approach could reveal more subtle brain alterations in the early stages of AD.

## Materials and Methods

### Subjects

We reviewed the Alzheimer's Disease Neuroimaging Initiative (ADNI) database and selected subjects with complete information, such as cerebrospinal fluid (CSF) biomarkers, 3D T1-weighted (T1w) images, rsfMRI images, and neuropsychological tests at the same time point. Based on the cutoff value of previous research on CSF biomarkers, the threshold of amyloid-β 1 to 42 peptide (Aβ_1−42_) is set at 192 ng/L, and the threshold of tau phosphorylated at the threonine 181 position (p-tau_181p_) is set at 23 ng/L (Shaw et al., 2009, 2011). After screening, the dataset contained 86 subjects, all from ADNI2.

According to the cutoff value and different cognitive stages, 11 CU individuals with the biomarker characteristic of “A–T–” were defined as healthy control (HC), 75 subjects with the biomarker characteristic of “A+T+” were further divided into three groups: 22 CU, 33 MCI, and 20 d-AD.

### CSF Analyses

Cerebrospinal fluid data were downloaded from the ADNI database. Baseline CSF samples were collected in the morning after an overnight fast and then processed, Aβ_1−42_ and p-tau_181p_ were measured subsequently as previously described (Shaw et al., 2009, 2011). In short, CSF was collected into collection tubes or syringes provided to each site, then, transferred into polypropylene transfer tubes within an hour after collection followed by frozen on dry ice, and transported overnight on dry ice to the ADNI Biomarker Core laboratory at the University of Pennsylvania Medical Center. These samples were thawed at room temperature (1 h) and gently mixed to prepare aliquots (0.5 ml). The aliquots were stored in bar code-labeled polypropylene vials at −80°C. Aβ_1−42_, t-tau, and p-tau_181_ were measured using the multiplex xMAP Luminex platform (Luminex Corp, Austin, TX, USA) with Innogenetics (INNO-BIA AlzBio3; Ghent, Belgium; for research use-only reagents) immunoassay kit-based reagents.

### Imaging Acquisition

All participants underwent 3.0 T MR scans. Sequences were acquired as follows: (1) 3D T1w magnetization prepared rapid gradient echo (MPRAGE) sequence (acquisition plane = SAGITTAL; flip angle = 9.0 degree; Matrix X = 256.0 pixels; Matrix Y = 256.0 pixels; Matrix Z = 170.0; pixel spacing X = 1.0 mm; pixel spacing Y = 1.0 mm; slice thickness = 1.2 mm; TE = 3.1 ms; TI = 0.0 ms; TR = 6.8 ms); (2) rsfMRI echo-planar imaging sequence (flip angle = 80.0 degree; Matrix X = 64.0 pixels; Matrix Y = 64.0 pixels; pixel spacing X = 3.3 mm; pixel spacing Y = 3.3 mm; time points = 140; number of slices = 48; slice thickness = 3.3 mm; TE = 30.0 ms; TR = 3,000.0 ms).

### Data Processing

Structural and functional MR images were processed using DPABISurf_V1.3 toolkit (http://rfmri.org/DPABISurf) and fMRIPrep 20.0.5 (https://fmriprep.org) (Esteban et al., 2019), which is based on Nipype 1.4.2 (https://github.com/nipy/nipype) (Gorgolewski et al., 2011).

#### Anatomical Data Preprocessing

The T1w image was corrected for intensity non-uniformity (INU) with N4BiasFieldCorrection (Tustison et al., 2010), distributed with ANTs 2.2.0 (Avants et al., 2008), and used as T1w-reference throughout the workflow. The T1w-reference was then skull-stripped with a Nipype implementation of the antsBrainExtraction.sh workflow (from ANTs), using OASIS30ANTs as the target template. Brain tissue segmentation of CSF, white matter (WM), and gray matter (GM) was performed on the brain-extracted T1w using fast (FSL 5.0.9) (Zhang et al., 2001). Brain surfaces were reconstructed using recon-all (FreeSurfer 6.0.1) (Dale et al., 1999), and the brain mask estimated previously was refined with a custom variation of the method to reconcile ANTs-derived and FreeSurfer-derived segmentation of the cortical gray matter of Mindboggle (Klein et al., 2017). Volume-based spatial normalization to one standard space (MNI152NLin2009cAsym) was performed through non-linear registration with antsRegistration (ANTs 2.2.0), using brain-extracted versions of both T1w reference and the T1w template. The following template was selected for spatial normalization: ICBM 152 Non-linear Asymmetrical template version 2009c (Fonov et al., 2009).

#### Functional Data Preprocessing

For the BOLD data of each subject, the following preprocessing was performed. The first 10 functional images volumes were discarded for the stabilization of the gradient magnetic field and the adaptation of the subjects need to take some time. A reference volume and its skull-stripped version were generated using a custom methodology of fMRIPrep. The BOLD reference was then co-registered to the T1w reference using bbregister (FreeSurfer) which implements boundary-based registration (Greve and Fischl, 2009). Head-motion parameters with respect to the BOLD reference (transformation matrices, and six corresponding rotation, and translation parameters) are estimated using mcflirt before any spatiotemporal filtering occurs (FSL 5.0.9) (Jenkinson et al., 2002). BOLD runs were slice-time corrected using 3dTshift from AFNI 20160207 (Cox and Hyde, 1997), and resampled onto the surface fsaverage5 for surface-based analysis (FreeSurfer reconstruction nomenclature) or onto the MNI152NLin2009cAsym space for volume-based analysis. Gridded (volumetric) resamplings were performed using antsApplyTransforms (ANTs), configured with Lanczos interpolation to minimize the smoothing effects of other kernels (Lanczos, 1964). Non-gridded (surface) resamplings were performed using mri_vol2surf (FreeSurfer). Automatic removal of motion artifacts using independent component analysis (ICA-AROMA) (Pruim et al., 2015) was performed on the preprocessed BOLD volumes. Several confounding time-series were calculated based on the preprocessed BOLD: framewise displacement (FD), DVARS (D referring to the temporal derivative of time courses, VARS referring to RMS variance over voxels), and three region-wise global signals (Power et al., 2012, 2014). Linear trends were then removed. The signals of the CSF, the WM, and the whole-brain were calculated using the CompCor (Behzadi et al., 2007) method and regressed out.

For more details of the pipeline, please refer the section corresponding to workflows in the documentation of fMRIPrep.

#### ALFF Analyses

The ALFFs analyses was based on the DPABISurf_V1.3 toolkit. The time series of each vertex on the surface or voxel in the 3d space was transformed into the frequency domain to obtain the power spectrum. The square root was then calculated at each frequency of the power spectrum and an average square root of 0.01–0.08 Hz at each vertex on the surface was obtained, considered as ALFF (Zang et al., 2007; Zou et al., 2008). The ALFF was then converted to a *Z* score by subtracting the global mean value and dividing it by the SD. Finally, we smoothed *Z* maps with a 10 mm full width at half maximum (FWHM) Gaussian kernel on the surface, or with a 6 mm FWHM Gaussian kernel on 3d volumes. Considering that dividing a classic frequency band into more precise sub-bands may better reflect specific brain activity changes, we further analyzed the frequency bands Slow-5 (0.01–0.027 Hz) and Slow-4 (0.027–0.073 Hz), which had been found related to GM neural oscillations and could detect changes in different cognitive stages (Zuo et al., 2010).

### Statistical Analysis

For demographic and clinical data, ANOVA was used to compare the age, education, neuropsychological tests, and CSF biomarkers among four groups in SPSS 26.0. A chi-square test was employed for gender distribution difference assessment. The significance level was set at *P* < 0.05.

Analysis of covariance was used to find out brain activity changes among the four groups using the DPABISurf_V1.3 toolkit. Age, gender, education, and mean FD were taken as covariates. Permutation test with 5,000 permutations was used to find significant clusters, and multiple comparisons were corrected by the family-wise error rate (FWER) method with threshold-free cluster enhancement (TFCE) (Smith and Nichols, 2009). The statistical threshold was set to *P* < 0.025, as statistical analyses were performed in both hemispheres. For volume-based analysis, *P* < 0.05 was used as a threshold.

Clusters that showed significant group differences were saved as regions of interest (ROIs). We extracted standardized ALFF (zALFF) values from all subjects using these ROIs. Then pair-wise *post-hoc* comparisons were made in SPSS 26.0 and we marked out a group with a significant difference between the two groups. Bonferroni correction was used to correct the *post-hoc* comparisons (*P* < 0.05/6).

Furthermore, to investigate the classification performance of regional zALFF values, we made receiver operating characteristic (ROC) curves and calculated areas under the curve (AUCs). In summary, we separately measured the accuracy of discriminating HC from the other three groups (CU vs. HC, MCI vs. HC, and d-AD vs. HC). The accuracy, sensitivity, and specificity for each classifier were calculated.

Finally, correlation analyses were used to measure the relationships among zALFF values and neuropsychological scales as well as CSF biomarkers. Bonferroni correction was used to correct the correlation analyses (*p* < 0.05/9).

## Results

### Demographic and Clinical Characteristics

The demographic and clinical characteristics of the four groups are summarized in Table 1. Two CU, one MCI, and six d-AD were excluded for excessive head movement (head motion more than 3 mm or 3 degrees). In the current study, 11 HC, 20 CU, 32 MCI, and 14 d-AD subjects were finally enrolled. There was no significant difference in age, education, and mean FD among the four groups (*P* > 0.05). Gender, CSF biomarkers, and neuropsychological tests showed significant differences between at least two groups (Table 1). In general, the CSF pathological changes in MCI and d-AD groups were more obvious, and the neuropsychological scales of the d-AD group were significantly worse than the other three groups.

**Table 1:** Demographics and clinical characteristics of the four groups.

**Demographic data**	**HC (*n* = 11)**	**CU (*n* = 20)**	**MCI (*n* = 32)**	**d-AD (*n* = 14)**	***P*-value**	***Post hoc***
Age (years)	75.35 ± 8.22	74.62 ± 6.11	71.48 ± 6.20	75.51 ± 4.06	0.098^a^	
Gender (male/female)	0/11	9/11	19/13	9/5	0.002^b^	
Education (years)	16.09 ± 3.42	16.15 ± 2.28	16.72 ± 2.29	15.57 ± 2.87	0.556^a^	
Mean FD (mm)	0.14 ± 0.08	0.12 ± 0.04	0.12 ± 0.05	0.13 ± 0.04	0.805^a^	
Aβ_1−42_ (CSF, ng/L)	230.73 ± 27.59	150.63 ± 25.54	138.80 ± 22.91	125.69 ± 19.27	<0.001^a^	HC > CU > d-AD, HC > MCI
P-tau_181p_ (CSF, ng/L)	16.76 ± 4.19	44.60 ± 14.81	54.18 ± 25.00	56.54 ± 30.29	<0.001^a^	CU, MCI, d-AD > HC
T-tau (CSF, ng/L)	49.65 ± 16.53	81.18 ± 35.60	114.34 ± 56.07^d^	136.45 ± 91.86	0.001^a^	MCI, d-AD > HC, CU
MMSE total score	29.45 ± 1.04	28.60 ± 1.96	27.88 ± 1.79	21.36 ± 3.65	<0.001^a^	HC > MCI > d-AD, CU > d-AD
Word immediate recall score	15.91 ± 3.59	13.56 ± 2.68^c^	9.19 ± 3.47	3.50 ± 2.50	<0.001^a^	HC, CU > MCI > d-AD
Word delayed recall score	14 ± 3.55	12.17 ± 3.00^c^	7.13 ± 3.39	1.21 ± 1.63	<0.001^a^	HC, CU > MCI > d-AD
TMT-A (s)	32.64 ± 9.22	35.72 ± 8.41^c^	34.84 ± 16.19	72.71 ± 43.67	<0.001^a^	d-AD > HC, CU, MCI
TMT-B (s)	61.82 ± 19.90	88.50 ± 39.14^c^	104.03 ± 60.51	198.14 ± 77.46	<0.001^a^	d-AD > MCI > HC, d-AD > CU
BNT score	28.91 ± 1.22	28.89 ± 1.02	27.28 ± 2.94	21.64 ± 7.15	<0.001^a^	HC, CU, MCI > d-AD

*Data are presented as mean ± SD. HC, healthy control; CU, cognitively unimpaired; MCI, mild cognitive impairment; AD, Alzheimer's disease; d-AD, AD with dementia; FD, framewise displacement; Aβ_1−42_, amyloid-beta42; CSF, cerebrospinal fluid; p-tau_181p_, tau phosphorylated at the threonine 181 position; t-tau, total tau; MMSE, Mini-Mental State Examination; TMT-A, Trail Making Test A; TMT-B, Trail Making Test B; BNT, Boston Naming Test*.

a *The p-value was obtained by one-way analysis of variance (ANOVA)*.

b *The p-value was obtained by a two-tail Fisher's exact test*.

c *There were two missing values in the CU group*.

d *There were two missing values in the MCI group*.

### ALFF Analyses Under Three Frequency Bands

**Figure 2:**
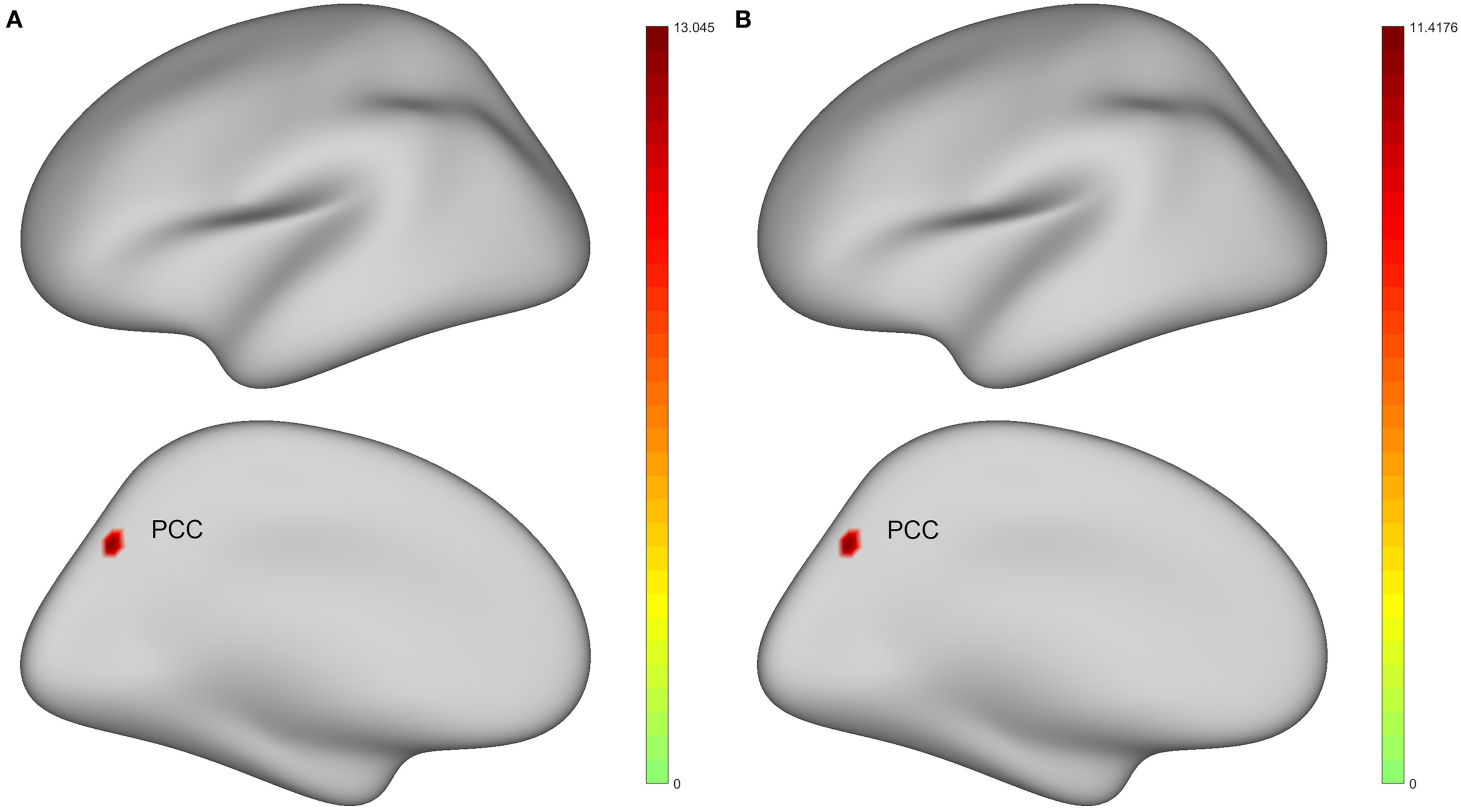
Brain regions with statistically significant differences in the standard amplitude of low-frequency fluctuations (zALFF) among healthy controls (HC), preclinical AD (CU), prodromal AD (MCI), and AD with dementia (d-AD). The results were obtained by analysis of covariance (ANCOVA) analysis after taking age, gender, education, and the mean framewise displacement (FD) as covariance [*P* < 0.025, permutation test number of 5,000, threshold-free cluster enhancement (TFCE), and family-wise error rate (FWER) correction]. **(A)** Under classic frequency band; **(B)** Under slow-4 band. Warmer color represents a more significant difference in statistical analysis. PCC, posterior cingulate cortex.

For surface-based analysis, ANCOVA analyses adjusting age, gender, education, and mean FD as covariates suggested that there were significant differences in spontaneous brain activity of left PCC among four groups under classic frequency band and slow-4 band. Figure 2 shows the brain regions where there are significant differences in brain spontaneous activity among the four groups under different frequency bands. Moreover, we added cluster size, MNI coordinates, peak value, and specific areas of the cerebral cortex [Human Connectome Project Multi-Modal Parcellation, HCP-MMP (Glasser et al., 2016)] to provide more detailed information (Table 2).

**Table 2:** Analysis of covariance (ANCOVA) results with age, gender, education, and the mean FD as covariates across healthy controls (HC), preclinical AD (CU), prodromal AD (MCI), and AD with dementia (d-AD).

**Frequency bands**	**Brain region**	**HCP-MMP**	**Cluster size (mm)**	**MNI coordinates**			**Peak value**
				**X**	**Y**	**Z**	
0.01–0.08 Hz	PCC	15	29.9787	27.5957	−72.7584	25.2395	13.045
0.027–0.073 Hz (Slow-4)	PCC	15	29.9787	27.3765	−72.9999	27.7724	11.4176

*Statistically significant differences in standard amplitude of low-frequency fluctuation (zALFF) value were defined as P < 0.025, permutation test number of 5,000, threshold-free cluster enhancement (TFCE), and family-wise error rate (FWER) corrected after taking age, gender, education, and the mean FD as covariance. HCP-MMP, Human Connectome Project Multi-Modal Parcellation; MNI, Montreal Institute of Neurology; PCC, posterior cingulate cortex*.

The *post-hoc* analyses found that after Bonferroni correction, the zALFF values of the left PCC in the d-AD group were significantly decreased compared with the other three groups under the classic frequency band, and the CU group had decreased zALFF compared with the HC group (Table 3). In the slow-4 band, there were significant differences among the other two groups except for that non-significant difference between the CU group and MCI group.

**Table 3:** The standard amplitude of low-frequency fluctuations (zALFF) values extracted from the left posterior cingulate cortex (PCC) in each group under classic frequency band and Slow-4 band (controlled for age, gender, education, and the mean FD).

	**HC**	**CU**	**MCI**	**d-AD**	***Post-hoc***
Classic frequency band	1.02 ± 0.62	0.48 ± 0.57	0.54 ± 0.53	−0.21 ± 0.38	HC > CU > d-AD, MCI > d-AD
Slow-4 band	1.12 ± 0.74	0.45 ± 0.57	0.51 ± 0.56	−0.20 ± 0.38	HC > CU/MCI > d-AD

*HC, healthy control; CU, cognitively unimpaired; MCI, mild cognitive impairment; AD, Alzheimer's disease; d-AD, AD with dementia*.

As for slow-5 band and volume-based analysis, there was no significant difference among the four groups.

### Classification

**Figure 3:**
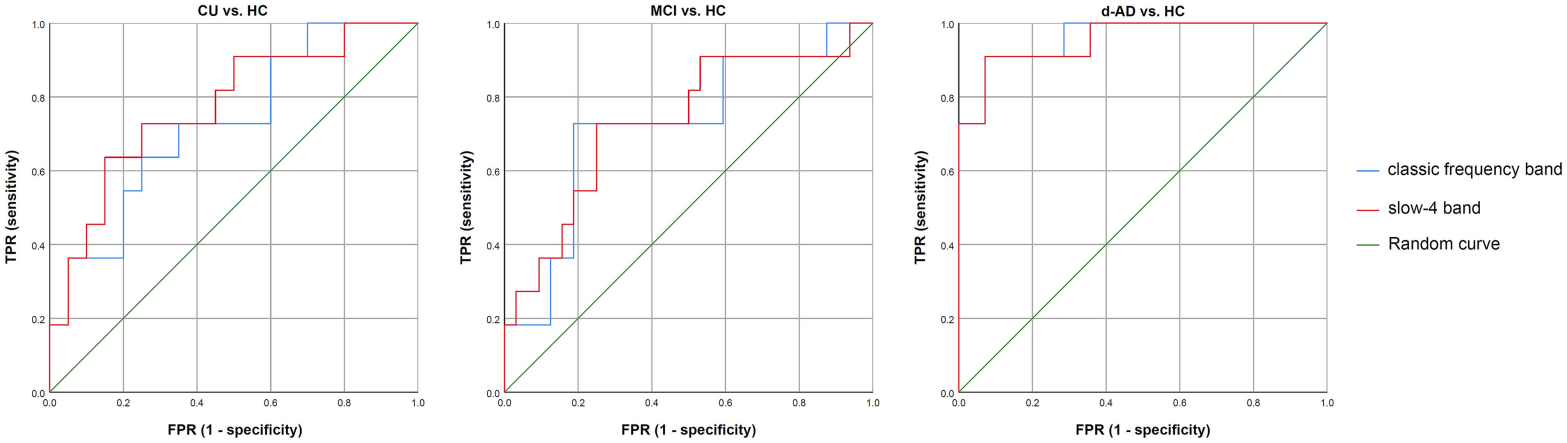
The classification performance and receiver operating characteristic (ROC) curves of CU vs. HC, MCI vs. HC, and d-AD vs. HC under classic frequency band and slow-4 band. HC, healthy control; CU, cognitively unimpaired; MCI, mild cognitive impairment; AD, Alzheimer's disease; d-AD, AD with dementia.

The classification performance and ROC curves are depicted in Figure 3. The AUC values for the classification of CU vs. HC, MCI vs. HC, and d-AD vs. HC with classic frequency band features were 0.727, 0.722, and 0.961, respectively, and with slow-4 band features were 0.773, 0.733, and 0.955, respectively. Meanwhile, the accuracy for the classification of CU vs. HC, MCI vs. HC, and d-AD vs. HC with classic frequency band features were 0.710, 0.791, and 0.920, respectively, and with slow-4 band features were 0.774, 0.744, and 0.920, respectively.

### Relationships With CSF Biomarkers and Neuropsychological Tests

We correlated zALFF values of the left PCC with CSF biomarkers and neuropsychological scales (Table 4). Under both classic frequency band and slow-4 band, the zALFF values of the left PCC had significant positive correlations with Aβ_1−42_, Mini-mental State Examination (MMSE) total score, word immediate recall score, word delayed recall score, and Boston Naming test (BNT) score, and significant negative correlation with p-tau_181p_, total tau (t-tau), Trail Making Test A (TMT-A), and Trail Making Test B (TMT-B).

**Table 4:** Correlation between zALFF values of the left PCC with cerebrospinal fluid (CSF) biomarkers and neuropsychological scales.

		**Aβ_1–42_**	**P-tau_181P_**	**T-tau**	**MMSE total score**	**Word immediate recall score**	**Word delayed recall score**	**TMT-A**	**TMT-B**	**BNT score**
Classic frequency band	*r*	0.343^**^	−0.236^*^	−0.315^*^	0.423^**^	0.354^**^	0.324^**^	−0.332^**^	−0.296^*^	0.398^**^
	*p*	0.002	0.039	0.006	<0.001	0.002	0.005	0.004	0.010	<0.001
Slow-4 band	*r*	0.381^**^	−0.247^*^	−0.316^*^	0.413^**^	0.356^**^	0.345^**^	−0.320^**^	−0.325^**^	0.384^**^
	*p*	0.001	0.030	0.006	<0.001	0.002	0.002	0.005	0.004	0.001

*Aβ_1−42_, amyloid-beta42; p-tau_181*p*_, tau phosphorylated at the threonine 181 position; t-tau, total tau; MMSE, Mini-Mental State Examination; TMT-A, Trail Making Test A; TMT-B, Trail Making Test B; BNT, Boston Naming Test*.

** *Refer to statistical significance after bonferroni correction*.

* *Refer to statistical significance before bonferroni correction*.

## Discussion

In this study, we used the surface-based approach and ALFF method to investigate spontaneous brain activity alterations in biological AD subjects. We found significantly different zALFF values in the left PCC region among the four groups, showing a decreasing gradient along with disease severity. Notably, CU subjects already had altered PCC activities, suggesting a very early effect of AD pathologies. Moreover, CSF biomarkers and neuropsychological tests showed significant correlations with zALFF values. Therefore, zALFF values of the left PCC may be a potential imaging marker for the early diagnosis of AD. Since there were no significant findings by the volume-based method, we suggest that a surface-based approach may increase the ability to detect such cortical function alteration.

We found significant differences in spontaneous brain activity of the left PCC region among the four groups. Further, *post-hoc* analyses showed that in the AD group the zALFF values of the left PCC decreased significantly compared with the other three groups, which is associated with CSF biomarkers and neuropsychological assessments, such as memory, language, and executive functions. PCC is the central part of the default mode network (DMN) associated with episodic memory retrieval. Moreover, it connects different subsystems of the DMN (Wagner et al., 2005; Buckner et al., 2008), which is a group of brain regions that support brain activity during resting state (Raichle et al., 2001) and plays a role in spontaneous cognition and functional balance with other brain systems (Raichle, 2015). As previous studies reported, cortical thinning, functional connectivity (FC) declining, glucose hypometabolism, and pathology aggregation in DMN areas have been observed in AD and MCI subjects or individuals at high risk of AD (Braak and Braak, 1991; Price and Morris, 1999; Dickerson et al., 2009; Gili et al., 2011; Brier et al., 2012; Hafkemeijer et al., 2012; Liguori et al., 2016; Lu et al., 2017). By using amyloid positron emission tomography (PET), early amyloid deposition in PCC and other regions in DMN have been detected in AD, MCI, and even at presymptomatic stages (Buckner et al., 2008; Johnson et al., 2014; Leech and Sharp, 2014). Meanwhile, most of the tau-dependent brain networks overlapped with ventral and dorsal DMN regions (Hoenig et al., 2018). The results corroborated previous findings of abnormal DMN in AD and its association with cognitive impairments.

It seems that the function of the left PCC is progressively impaired during the AD continuum and may serve as a stable imaging marker for monitoring the development of AD. Wang et al. (2011) reported that PCC showed the most significant ALFF difference among the AD, MCI, and healthy elderly groups. While ALFF values decreased in both AD and MCI groups, and the difference was only significant between patients with AD and healthy elderly (Wang et al., 2011). Similarly, Liang et al. (2014) found PCC had significant differences in ALFF values, and the trend of ALFF values was AD < late MCI < early MCI < NC. Most encouragingly, with a surface-based approach, in this study, we additionally found that ALFF values of left PCC were decreased in the CU group when the subjects had not shown any cognitive deficits. Further, using this index to classify CU and HC, the accuracy was 0.710. This may indicate that damage to the PCC began very early, possibly due to Aβ deposition (Johnson et al., 2014), and PCC ALFF values may have great potentials for early diagnosis. Notably, the volume-based analysis showed no significant difference between the four groups using the same statistical threshold. As previously proposed, the surface-based method would have better repeatability and sensibility than the volume-based approach. Also, it can provide additional guarantees for more spatially specific clusters (Oosterhof et al., 2011; Tucholka et al., 2012). Thus, we suggest that a surface-based approach would be beneficial for the detection of early cortical function alterations in AD.

It is worth noting that we only found changes in the left but no right PCC. In previous works of literature, results of laterality were indeed more common. The most reliable and sturdy metabolic changes for predicting conversion from aMCI to AD were hypometabolism in the left PCC/precuneus (Ma et al., 2018). In brain 18F-fluorodeoxyglucose (FDG) PET, a considerable number of patients with AD showed hemispheric asymmetries of hypometabolism (Murayama et al., 2016). Neuropathological studies have also shown that the left hemisphere was more likely to suffer from AD-related neurodegeneration than the right hemisphere (Janke et al., 2001). Also, in other fields, researchers have found that in primary progressive aphasia patients, atrophy, neuron loss, and disease-specific proteinopathy were more severe in the language-dominated hemispheres (Mesulam et al., 2014). Concerning most of the subjects who were right-handed, it is possible that increased intensity and frequency of usage of the left hemisphere resulted in faster AD-related neurodegeneration.

Among the three frequency bands, we found similar results in the classic frequency band and Slow-4 band but not the Slow-5 band. The previous studies on these three bands of AD have shown that the test-retest reliability of Slow-4 was greater and more widely distributed than that of Slow-5. In amnestic MCI, ALFF values in the left hippocampus and PCC were significantly reduced under the slow-4 band (Zhao et al., 2015). Compared with HC, ALFF values in the right putamen of MCI decreased in the slow-4 band (Ren et al., 2016). The Slow-4 band may be more sensitive to changes in AD pathologies. Furthermore, compared with the classic frequency band, zALFF values in the Slow-4 band additionally showed a statistical difference between the HC and MCI groups, suggesting that ALFF analysis within this frequency band could be more stable.

The current study is subject to limitations. First, the sample size was relatively small, and all the members of the control group were women. Although ADNI is the largest AD database and contains over a thousand samples, subjects with both rsfMRI scan and CSF biomarkers in the ADNI2 dataset are not common. Nevertheless, we found consistent changes in the left PCC, suggesting the robustness of the current method. Second, the research only focused on cross-sectional analysis, a longitudinal study is needed to fully demonstrate the progressive impairment of PCC function. Third, we only used one approach (ALFF) to find imaging markers. Future studies combining multi-parametric maps and multi-voxel pattern analysis methods may produce increased sensitivity for recognizing preclinical AD (Premi et al., 2016). Finally, we had not included PET biomarkers due to the scarcity of tau PET data. As CSF biomarkers could only reflect the pathological state but not pathological deposition in specific regions (Jack et al., 2018), further studies are needed for understanding the relationship between local brain activity changes and regional pathological accumulation.

In conclusion, we used a surface-based approach combined with specific frequency band analysis to explore the alterations of spontaneous brain activity in different stages of biologically defined patients with AD. We found consistently decreased ALFF in the left PCC of biological AD, even in preclinical AD when cognitive functions were relatively normal. It is the potential to be an early imaging marker for AD diagnosis and disease progression monitoring. Furthermore, with the continuous development of automatic and fast analytical methods, easier clinical access to this marker could be expected.

## Data Availability Statement

The original contributions presented in the study are included in the article/supplementary material, further inquiries can be directed to the corresponding author/s.

## Ethics Statement

The studies involving human participants were reviewed and approved by Alzheimer's Disease Neuroimaging Initiative. The patients/participants provided their written informed consent to participate in this study.

## Author Contributions

PH, YY, and ZZ designed the study. ZZ and QZ collected clinical and MRI data. ZZ and LK analyzed the MRI data and wrote the protocol. QZ, XL, KL, XX, MZ, YY, and PH assisted with the organization of results and interpretation of findings. ZZ wrote the first draft of the manuscript. PH revised the manuscript. All authors contributed to the article and approved the submitted version.

## Conflict of Interest

The authors declare that the research was conducted in the absence of any commercial or financial relationships that could be construed as a potential conflict of interest.

## Publisher's Note

All claims expressed in this article are solely those of the authors and do not necessarily represent those of their affiliated organizations, or those of the publisher, the editors and the reviewers. Any product that may be evaluated in this article, or claim that may be made by its manufacturer, is not guaranteed or endorsed by the publisher.
